# The Interrelationship of Benefit Finding, Stigma, and Suicide Risk Among Patients with Schizophrenia and Their Caregivers: A Six-Month Follow-Up Study

**DOI:** 10.3390/healthcare12212176

**Published:** 2024-10-31

**Authors:** Heng Lee, Pin-Han Peng, Nien-Mu Chiu, Yu-Chi Huang, Pao-Yen Lin, Chi-Fa Hung, Yu Lee, Liang-Jen Wang

**Affiliations:** 1 Department of Medical Education, Chang Gung Memorial Hospital, Keelung 20401, Taiwan; lhps09@gmail.com; 2 Department of Psychiatry, Kaohsiung Chang Gung Memorial Hospital and Chang Gung University College of Medicine, Kaohsiung 83301, Taiwan; pengpinhan@gmail.com (P.-H.P.); neinmuch@adm.cgmh.org.tw (N.-M.C.); ychuang01@gmail.com (Y.-C.H.); paoyenlin@gmail.com (P.-Y.L.); chifa.hung@gmail.com (C.-F.H.); 3 Department of Child and Adolescent Psychiatry, Kaohsiung Chang Gung Memorial Hospital and Chang Gung University College of Medicine, Kaohsiung 83301, Taiwan

**Keywords:** benefit finding, caregivers, stigma, suicide, schizophrenia

## Abstract

Objective: This study aimed to assess the dyadic dynamics of benefit finding, stigma, and suicide risk on the depression severity of individuals with schizophrenia and their caregivers. Methods: We prospectively recruited a total of 72 individuals with schizophrenia and 72 caregivers of individuals with schizophrenia from a medical center in Taiwan between August 2022 and July 2023. Patients with schizophrenia and their caregivers were assessed using the Taiwanese Depression Questionnaire, Benefit Finding Scale, Explanatory Model Interview Catalogue, Suicide Assessment Scale, and Mini International Neuropsychiatric Interview. Results: The most prevalent psychiatric diagnoses in the caregivers were depressive disorders (29.2%). Using structural equation modeling, we found that patients’ suicidal risk (β = 0.45, *p* < 0.001) may contribute to the severity of depression in individuals with schizophrenia. We also found that caregivers’ BF degree (β = −0.25, *p* < 0.01) and suicidal risk (β = 0.64, *p* < 0.001) may contribute to the severity of depression in the caregivers of individuals with schizophrenia. Furthermore, we found that caregivers’ suicidal risk (β = 0.28, *p* < 0.05) and caregivers’ stigma (β = −0.31, *p* < 0.01) level may contribute to the severity of depression in individuals with schizophrenia. Discussion: Clinicians should actively manage caregivers’ stigma and provide positive reinforcement for caregivers’ BF, as this may help reduce depression in both caregivers and individuals with schizophrenia. Additionally, assessing suicide risk in both groups is essential for preventing suicides among individuals with schizophrenia and caregivers alike.

## 1. Introduction

Schizophrenia is a severe mental disorder that has an intense impact on both the affected individual and society. McGrath and colleagues (2008) estimated a point prevalence of 4.6 per 1000 and a lifetime morbid risk of approximately 7 per 1000 [1]. Over 50% of the people who receive a schizophrenia diagnosis experience long-term psychiatric problems, and a schizophrenia diagnosis was also associated with poor physical condition and found to reduce life expectancy by 10–20 years [2].

Epidemiologic studies have shown that the prevalence of depression is high among people with schizophrenia, with the reported prevalence estimates of depression ranging between 7 and 65% depending on the study [3]. According to several studies, comorbid depression in patients with schizophrenia is correlated with an increased risk of suicide, frequent relapse and hospitalization, poor physical health, and poor treatment outcomes when compared to individuals with schizophrenia without depression [4,5].

The caregivers of individuals with schizophrenia face various challenges to their psychological and social well-being, thus being one is linked to a higher likelihood of experiencing psychological distress, anxiety, and depression [6]. Prasad et al., (2024) systemically reviewed 24 studies on depression in the caregivers of patients with schizophrenia and found that the prevalence of depression in samples of caregivers ranged between 12 and 40% [7]. Therefore, healthcare professionals should also pay attention to the mood state of caregivers of persons with schizophrenia.

Benefit finding (BF) can be conceptualized as looking for positive life changes or benefits from negative experiences such as chronic illness or trauma [8]. Evidence supporting BF has been seen in studies of a patient with diabetes mellitus [9] and patients with cancer, e.g., breast cancer [10], head and neck cancer [11], etc. Lee et al., (2017) investigated 148 Chinese early-stage breast cancer patients to examine the relationship between non-disclosure and depressive symptoms and found that a higher level of non-disclosure was associated with more depressive symptoms. The influence of non-disclosure was minimized among women with a higher level of BF [10]. Currently, no study has been performed on BF among patients with schizophrenia or their caregivers. Therefore, advanced studies are needed to explore BF on depression among individuals with schizophrenia and their caregivers.

Stigma refers to the stereotypes or negative perceptions linked with a person or group of people who are thought of as different from or even inferior to societal norms [12]. A meta-analysis study performed by Gerlinger et al., (2013) among 54 studies found that neighbors’ negative perception affects a significant number of individuals with schizophrenia. According to the authors, the perceived or experienced stigma is associated with more severe depressive symptoms, greater social anxiety and avoidant behavior, lower self-confidence, lower quality of life, and poorer social functioning [13]. Nimbhalkar et al., (2024) investigated 60 patients with schizophrenia and found that 23.3% of patients experienced severe stigma and that stigma is associated with lower level of functioning [14]. To date, dyadic studies regarding stigma among the caregivers of individuals with schizophrenia are scarce, so conducting studies to improve the understanding of the caregivers’ and patients’ feelings of stigma and to try to work through this dilemma is necessary.

Suicidal behavior in schizophrenia is an underestimated issue, with 25–50% of patients attempting suicide in their lifetime [15]. Lyu et al., (2021) conducted a study on the characteristics and risk factors for suicide in people with schizophrenia in comparison to those without schizophrenia. They found that the suicide group of schizophrenia patients was more likely to be female, older, suffer from chronic disease, suffer other psychiatric disorders, and have a family history of psychiatric disorders compared to those not diagnosed with schizophrenia [16].

The study by Huang et al., (2018) found that nearly one-fifth (18.8%) of caregivers for patients with mental disorders had experienced suicidal thoughts [17]. However, no studies have specifically addressed suicide risk in the caregivers of patients with schizophrenic disorder. Further studies on this topic are warranted. From the literature review mentioned above, we found that individuals with schizophrenia and their caregivers experience a high prevalence of depression. Patients with schizophrenia also face a significant risk of suicide and encounter high levels of stigma.

Very few studies have examined the relationship between BF, stigma, and suicide risk among patients with schizophrenia and their caregivers. The aim of this study was to investigate how BF, stigma, and suicide risk interact and affect the severity of depression in both individuals with schizophrenia and their caregivers. Gaining insight into the connections between BF, stigma, and suicide risk in the context of their interactions could offer a more holistic understanding of how to treat depression in individuals with schizophrenia and their caregivers, ultimately leading to reduced morbidity, improved quality of life, and lower mortality rates.

## 2. Materials and Methods

### 2.1. Participants

This study was performed using a cross-sectional, purposive sampling design. We recruited participants from the psychiatric outpatient clinic or psychiatric ward of a medical center from August 2022 to July 2023. The patient inclusion criteria were as follows: (1) aged over 18 y/o; (2) diagnosed with schizophrenia by staff psychiatrists according to the DSM-5 diagnostic system; and (3) the ability to understand the study procedure and provide written informed consent. The patient exclusion criteria were as follows: (1) a diagnosis of delirium, dementia, psychotic disorder due to another medical condition, substance/medication-induced psychotic disorder, schizoaffective disorder, delusional disorder, or bipolar disorder and (2) unable to complete the questionnaire or clinical interview.

The inclusion criteria for caregivers were as follows: (1) aged over 18 y/o; (2) patient’s principal caregivers, which is defined as “family members who live with the patients and take care of their daily needs”; and (3) the ability to understand the study procedure and provide written informed consent. Caregivers were excluded if they were unable to complete the questionnaire or clinical interview.

We had a total of 97 individuals with schizophrenia and 97 caregivers that finished our questionnaires at baseline and 72 pairs of individuals with schizophrenia and their caregivers that completed questionnaires during the 6-month follow-up period, for a 74.2% follow-up rate. Of those, 38 (52.8%) of the caregivers were parents, 18 (25%) were spouses, 9 (12.5%) were siblings, 6 (8.3%) were children, and 1 (1.4%) was a friend. The caregivers in our study have caring experience ranging from 1 to 41 years.

### 2.2. Assessments

#### 2.2.1. Numeric Pain Rating Scale (NPRS)

The Numeric Pain Rating Scale (NPRS) is a unidimensional measure in adults, including those with chronic pain, in order to assess pain intensity [18]. This scale uses an 11-point format ranges from “0”, which indicates one pain extreme (e.g., “no pain”), to “10”, which indicates the other pain extreme (e.g., “as bad as you can imagine”). A study was conducted to evaluate the NPRS’s reliability and found good reliability for both worst pain (r = 0.93) and average pain (r = 0.78) [18].

#### 2.2.2. Family APGAR Index

The family APGAR index was designed to evaluate an individual’s perception of family function. Five dimensions of family function were identified, adaptation, partnership, affection, growth, and resolve. The total scores range from 0 to 10, with higher scores representing superior family functioning [19]. The Chinese version was translated and validated by Chen et al. in 1980 and showed acceptable validity [20].

#### 2.2.3. Taiwanese Depression Questionnaire (TDQ)

The TDQ, a culturally sensitive self-administered instrument, was developed to screen possible depression cases in Taiwan [21]. This questionnaire is composed of 18 items, including mood, sleep problem, appetite, fatigue/weakness, interest, pessimism, somatic symptoms, etc. Individuals are asked to specify whether they have experienced each item using a 4-point Likert scale, where the total score ranges from 0 to 54. We also completed a study to assess the validity of the TDQ in detecting depression among cancer patients, which yielded fair validity [22].

#### 2.2.4. Big Five Inventory-10 (BFI-10)

The BFI-10 was created to assess personality in one minute. This 10-item version of the Big Five Inventory (BFI-44) has scores that range from 0 to 10 and accounts for about 70% of the variance found in the full-scale BFI-44. It has shown good structural validity, convergent validity, external validity, and test–retest reliability [23].

#### 2.2.5. Brief Life Event Questionnaire (LTEQ)

The LTEQ is a brief evaluation tool commonly used to assess stressors in epidemiologic studies [24]. Developed by Brugha and Cragg, the LTEQ is a 12-yes-or-no-question self-administered questionnaire for evaluating stressful life events. It is especially well-suited for psychosocial research [25]. The total scores on the LTEQ range from 0 to 15, with higher scores indicating a greater level of stress.

#### 2.2.6. Benefit Finding Scale (BFS)

The Benefit Finding scale (BFS), developed by Tomich and Helgeson, was designed for breast cancer patients to assess the perception that positive contributions were made to one’s life by the experience of being diagnosed with and treated for breast cancer [26]. The Chinese version of the BFS demonstrated good patient acceptability, as well as satisfactory convergent validity, discriminant validity, concurrent validity, and internal consistency among Chinese patients with early-stage cancer [27]. Each of the 22 items ranged from 1 (not at all) to 5 (extremely) on a five-point Likert scale [27]. The total scores on the BFS range from 5 to 110, with higher scores indicating greater levels of benefit finding.

#### 2.2.7. Stigma Subscale of the Explanatory Model Interview Catalogue (EMIC)

The Explanatory Model Interview Catalogue (EMIC) is a semi-structured interview tool grounded in anthropology that systematically investigates patients’ help-seeking behaviors, providing both quantitative and qualitative data [28]. Over the past 20 years, EMIC has served as a key research instrument in cultural psychiatry, centering on patients’ experiences of illness behavior and stigma [29]. The Stigma Subscale of the EMIC has a possible total score ranging from 0 to 24, with higher scores reflecting greater levels of stigma.

#### 2.2.8. Suicide Assessment Scale (SAS)

The SAS was created by a prospective study of patients with a history of repeatedly attempted suicide within a general hospital setting [30]. Having fair reliability and validity, the SAS was suggested to assess patients’ suicidal risk in clinical or research settings [31]. Negative ideation, positive ideation, impulsivity, and aggression represent the four dimensions of the SAS. The total scores on the SAS range from 0 to 80, a higher score indicates a higher degree of suicidal risk [31].

#### 2.2.9. Mini International Neuropsychiatric Interview (MINI)

The MINI is a concise, structured interview created to provide precise psychiatric diagnoses [32]. The validity and reliability of the MINI have been compared with the Structured Clinical Interview for DSM-IIIR Patients (SCID-P), yielding fair results [33]. The interview typically takes about 15 to 20 min to complete. The Suicidal Scales of the MINI consist of six items, which cover aspects such as suicidal thoughts, planning, and actions.

### 2.3. Procedures

The study procedures were as follows: (1) The in-charge doctor referred potential candidates, including patients with schizophrenia and their caregivers, from psychiatric clinical settings such as outpatient clinics or psychiatric wards. A research assistant then contacted them to explain the study’s aims and procedures in detail to obtain their informed consent. (2) Both the individuals with schizophrenia and their caregivers were given the NPRS, BFS, Stigma Scale of the EMIC, TDQ, SAS, Suicidal Scales of the MINI, LTE-Q, BFI-10, and Family APGAR Index. (3) The MINI was performed by one staff psychiatrist to achieve psychiatric diagnoses. (4) Our trained research assistant gathered demographic and clinical data from each patient and caregiver using the aforementioned instruments through face-to-face interviews. (5) At the second visit (month 6), the above questionnaires were completed by a research assistant and one staff psychiatrist.

### 2.4. Statistical Analyses

Descriptive and inferential statistics were analyzed using SPSS for Windows v. 24.0. Chi-square and Student’s *t* tests were used to examine the differences in demographic data and then test the clinical characteristics of individuals with schizophrenia and their caregivers. A Pearson correlation analysis was conducted to examine our hypothesis that patients’ BF, stigma, suicide risk, and depression are correlated with those of their caregivers. To determine the impact of BF, suicide risk, and stigma on patients’ and caregivers’ depression, we demonstrated actor and partner effects by using the actor–partner interdependence model (APIM) with a dyads regression model [34]. The APIM was assessed using structural equation modeling (SEM), which was analyzed using SPSS Amos 24.0 [35]. SEM is a multivariate, hypothesis-driven method that uses a structural model to illustrate the causal relationships among multiple variables. We conducted sample size calculations for the SEM. According to Bentler’s recommendations, 5 to 10 observations are needed for each estimated parameter in SEM. Since our study has 8 parameters, a sample size of 40 to 80 would be sufficient [36].

## 3. Results

Of the 72 individuals with schizophrenia who successfully completed the study, 68.1% (n = 49) were female. The average age of the individuals with schizophrenia was 48.1 ± 11.1 years, with a mean educational level of 12.5 ± 3.5 years. Most were unmarried (69.4%) and unemployed (80.6%). The average duration of the illness was 19.1 ± 10.4 years (Table 1). Among the 72 caregivers who completed the study, 59.7% (n = 43) were female. The average age of the caregivers was 60.4 ± 12.8 years, and their mean educational level was 12.4 ± 3.3 years. Most caregivers were married (69.4%) and 59.7% were unemployed. The average duration of caregiving was 16.6 ± 9.5 years (Table 1).

Compared to their caregivers, patients with schizophrenia were younger (48.1 ± 11.1 vs. 60.4 ± 12.8, t = −6.16, *p* < 0.001), more likely to be unmarried (69.4% vs. 30.6%, χ^2^ = 21.78, *p* < 0.001), and more likely to be unemployed (80.6% vs. 59.7%, χ^2^ = 7.46, *p* < 0.05). They also had higher rates of personal psychiatric history (98.6% vs. 16.9%, χ^2^ = 99.01, *p* < 0.001), suicide attempts history (27.8% vs. 2.8%, χ^2^ = 17.38, *p* < 0.001), and anxiolytic/hypnotic use (69.4% vs. 16.7%, χ^2^ = 40.90, *p* < 0.001), while reporting lower family psychiatric history (χ^2^ = 10.12, *p* < 0.01). Patients had higher APGAR scores (8.3 ± 3.0 vs. 7.1 ± 3.2, t = 2.34, *p* < 0.05), lower extraversion scores on the BFI-10 (4.5 ± 2.0 vs. 5.3 ± 1.9, t = −2.37, *p* < 0.05), and lower conscientiousness scores (6.6 ± 1.8 vs. 7.6 ± 1.6, t = −3.58, *p* < 0.001) but higher neuroticism scores (6.6 ± 1.9 vs. 5.2 ± 1.7, t = 4.76, *p* < 0.001). Patients also scored lower on the BFS total (53.2 ± 20.2 vs. 64.9 ± 21.7, t = −3.36, *p* = 0.001), including subscales for acceptance (8.1 ± 3.7 vs. 9.4 ± 3.5, t = −2.24, *p* < 0.05), family relations (5.1 ± 2.3 vs. 6.2 ± 2.2, t = −2.79, *p* < 0.05), worldview (8.3 ± 4.1 vs. 11.7 ± 4.3, t = −4.80, *p* < 0.001), and personal growth (15.1 ± 7.4 vs. 20.3 ± 8.1, t = −3.98, *p* < 0.001). Additionally, patients reported higher scores on the Stigma Scale of the EMIC (14.0 ± 11.3 vs. 9.1 ± 7.9, t = 3.03, *p* < 0.05) and lower impulsivity scores on the SAS (1.1 (0–11) vs. 2.0 (0–15), t = −2.14, *p* < 0.05) compared to their caregivers (Table 1).

In addition to all patients having schizophrenia as their primary psychiatric diagnosis, the most common secondary diagnoses among the patients were other specified depressive disorder (2.8%) and obsessive–compulsive disorder (2.8%) (Table 2).

Among caregivers, depressive disorders were the most prevalent, affecting 29.2% of them, with other specified depressive disorders being the most common subtype (18.1%). Generalized anxiety disorder was the most frequent anxiety disorder, affecting 8.3% of caregivers. Overall, 43.1% of caregivers had at least one psychiatric diagnosis (Table 2).

Pearson correlation analysis revealed significant correlations between patients’ BF, stigma, suicide risk, and depression and those of their caregivers (Table 3).

**Figure 1:**
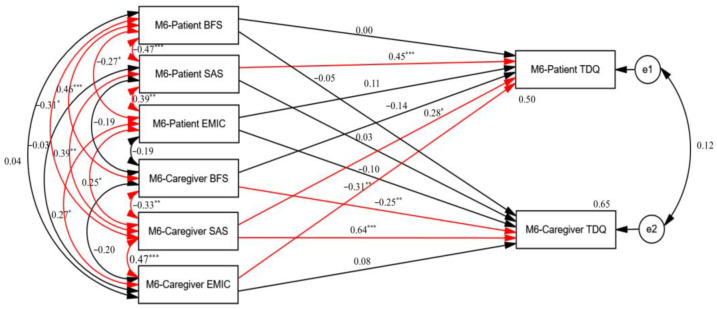
Structural equation modeling (SEM) of factors linked to TDQ scores in schizophrenia patients and their caregivers at 6-month follow-up. Model summary: chi-square = 0; df = 0; *p* = \p. The model fit: AGFI = \AGFI; RMSEA = \RMSEA; AIC = 72.00. * *p* < 0.05; ** *p* < 0.01; *** *p* < 0.001; the red line indicates *p* < 0.05.

Structural equation modeling (SEM) showed that patients’ suicidal risk (β = 0.45, *p* < 0.001) significantly contributed to the severity of depression in individuals with schizophrenia (Figure 1). Caregivers’ BF level (β = −0.25, *p* < 0.01) and caregivers’ suicidal risk (β = 0.64, *p* < 0.001) significantly contributed to the severity of depression in the caregivers of individuals with schizophrenia (Figure 1). Additionally, caregivers’ suicidal risk (β = 0.28, *p* < 0.05) and caregivers’ stigma intensity (β = −0.31, *p* < 0.01) significantly contributed to the severity of depression in individuals with schizophrenia (Figure 1). Moreover, an interactive relationship was found in the BF levels of individuals with schizophrenia and their caregivers (β = 0.46, *p* < 0.001), stigma intensity (β = 0.27, *p* < 0.05), and suicidal risk (β = 0.39, *p* < 0.01) (Figure 1).

## 4. Discussion

To establish models of the possible mechanisms underlying the connection between related factors and depression in patients with schizophrenia and their caregivers, we used SEM to examine APIM. The actor effects were as follows: (1) patient suicide risk was significantly linked with the depression severity of individuals with schizophrenia; (2) caregiver suicide risk and caregiver BF were significantly linked with the depression severity of the caregivers. The following partner effects were also found: (1) the caregivers’ suicide risk (β = 0.28, *p* < 0.05) and stigma (β = −0.31, *p* < 0.01) were significantly linked with the depression severity of individuals with schizophrenia; (2) the patients’ stigma severity/BF level/suicidal risk and their caregivers’ stigma severity/BF level/suicidal risk had significant interactive effects, respectively.

To the best of our knowledge, this is the first prospective study to investigate the potential interrelationship between benefit finding, stigma, and suicide risk among patients with schizophrenia and their caregivers. While there are two studies involving the patient–caregiver dyad, one examined the relationship between beliefs about psychotropic medications, their side effects [37], and adherence and the other focused on mutuality and quality of life [38]. Hsiao et al., (2021) explored the dyadic relationship between mutuality and health-related quality of life in patients with schizophrenia and their caregivers. Among 133 dyads, both patients’ and caregivers’ mutuality were linked to their own health-related quality of life (actor effect), as well as their partners’ health-related quality of life (partner effect) [38]. No studies have addressed a topic similar to ours, highlighting the need for further research to confirm our findings.

The suicidal risk in both individuals with schizophrenia and their caregivers impacts depression severity. A previous study showed that post-psychotic depression is one of the associated factors of suicide among patients with schizophrenia [16]. Our prior study also found that the severity of suicide risk is one of the associated factors of depression in patients with Parkinson’s disease [39]. The afore-mentioned studies support our result that the suicide risk severity of schizophrenia may impact the severity of depression among individuals with schizophrenia.

In this study, caregivers’ BF was significantly linked with the caregivers’ depression severity. In our previous study on PD patients and their caregivers, among 120 pairs of PD patients and caregivers, we found that caregivers’ BF was significantly negatively linked with their depression severity [40]. In another study from China, Wen et al. investigated 228 esophageal cancer patients’ caregivers and found that the caregivers’ BF played a mediating role in the anxiety/depression of the caregivers, as well as that a higher level of BF may lower the psychological distress of caregivers, which could lead to a lower severity of depression [41]. These studies support our result that caregivers’ BF may inversely impact caregivers’ depression.

In this study, we found that caregivers’ stigma level was negatively linked with the depression severity of individuals with schizophrenia. One possible explanation for this finding is that caregivers may conceal their stigma, which may impact the depression of individuals with schizophrenia.

It is intriguing that caregivers’ suicidal risk was significantly linked with the depression severity of individuals with schizophrenia. We can hypothesize that even though individuals with schizophrenia have impaired cognition, they can still recognize the threat of caregivers’ suicidal risk, which may impact patients’ depression. More studies are warranted to confirm our above findings.

We observed three interactive partner effects: the patients’ stigma severity/BF level/suicidal risk and their caregivers’ stigma severity/BF level/suicidal risk all had significant interactive effects, respectively. Lin et al., (2021) explored the dyadic relationship of BF and its impact on quality of life in colorectal cancer survivor and spousal caregiver couples, finding a positive correlation between the BF levels of both patients and their caregivers [42]. Similarly, Chou et al., (2024) studied 120 pairs of patients with Parkinson’s disease (PD) and their caregivers, discovering that the BF levels of PD patients and their caregivers influenced each other [40]. Both of these studies support our finding that the BF levels of individuals with schizophrenia and their caregivers also have an interactive effect.

In our earlier study focusing on PD patients and their caregivers, we identified a positive correlation between the stigma experienced by PD patients and that reported by their caregivers. Consequently, we propose that PD symptoms are not only linked to patient stigma but also influence caregiver stigma, which may help explain the observed positive relationship between the stigma levels of PD patients and their caregivers [37]. This finding aligns with our observation that the severity of stigma in individuals with schizophrenia and their caregivers is interrelated.

It should also be noted that, in our study, the suicidal risks of individuals with schizophrenia and their caregivers had an interactive effect. As mentioned in an earlier section, caregivers’ suicidal risk impacted the depression severity of individuals with schizophrenia. Moreover, caregivers’ suicidal risk had an influence on schizophrenic suicidal risk, even with patients’ declining cognition. Likewise, we found that the suicidal risk of individuals with schizophrenic impacted the suicidal risk their caregivers. This interaction is very important in clinical settings, and clinicians should carefully evaluate and rigorously manage the suicidal risks of individuals with schizophrenia and their caregivers.

In this study, we found that patients with schizophrenia perceive more family support, more neurotic personality, impaired BF degrees, and higher stigma levels than their caregivers. This result suggests that caregivers of individuals with schizophrenia provide substantial support to patients, have more normal personality traits, embody strong positive values to face this chronic disease, and cope well with this disease in order to experience less stigmatization than schizophrenia patients. Few previous studies have compared psychological wellbeing, e.g., family support, personality attributes, BF, and stigma, among individuals with schizophrenia and their caregivers. Chou et al., (2024) investigated 120 pairs of patients with Parkinson’s disease (PD) and their caregivers and found that PD patients have higher stigma levels and lower BF degrees than their caregivers, which was in line with our results [40]. Together, these two studies suggest that patients may have a greater stigma level and lower BF than their caregivers regardless of the chronic physical disease or mental disorder. Further studies should be conducted to compare the afore-mentioned stigma and BF among patients with mental disorders/physical diseases and their caregivers.

The most prevalent psychiatric diagnoses among the caregivers of individuals with schizophrenia were depressive disorders (29.2%). Apart from major depressive disorder (8.3%), the majority of depressive caregivers had other specified depressive disorders (18.1%) and persistent depressive disorder (2.8%). This result suggests that the depressive caregivers of individuals with schizophrenia had been taking care of patients for a long time and thus were vulnerable to chronic minor depressive states. Compared to caregivers for other diseases, the prevalence of depression in this study is higher than that for depressive disorder (25.8%) [43], head and neck cancer (14.7%) [44], and Parkinson’s disease (PD) (11.1%) [39]. This finding suggests that when caring for an individual with schizophrenia, the risk of having depression is higher than both physical diseases and depressive disorder, which may be due to the greater burden of providing more psychosocial care.

This study has several strengths. 1. It is the first prospective study to explore the potential interrelationship between BF, stigma, and suicidal risk among patients with schizophrenia and their caregivers using APIM. 2. This may be the first paper to discuss the interrelated suicide risk of patients with schizophrenia and their caregivers. No prior studies have examined this finding. However, there are also several limitations to this study worth noting. First, all patients were recruited from a single general hospital, and the sample size may be not large enough to accurately reflect the general population. Second, our study utilized purposive sampling, which could introduce sampling bias. Third, various tools could be employed to assess BF, stigma, and suicidal risk, potentially leading to discrepancies when comparing our results with those of other studies. Fourth, While the SEM analysis suggests that the suicide risk in patients with schizophrenia may be linked to the severity of their depression, this phenomenon could also arise from the relationship between depression severity and suicide risk. In this study, the causal relationship between suicide and depression remains uncertain.

## 5. Conclusions

The clinical implications of this study are: (1) The suicidal risk of individuals with schizophrenia may contribute to their depression severity. (2) Caregivers’ BF and the suicidal risk of schizophrenic caregivers may contribute to their depression severity. (3) The suicidal risk and stigma of caregivers may contribute to the depression severity of patients with schizophrenia. (4) There were significant interactive effects between the BF levels, stigma, and suicidal risk of individuals with schizophrenia and their caregivers. These findings indicate that clinicians should actively manage caregivers’ stigma (e.g., through psychoeducation, promoting open dialogue, and highlighting recovery stories) and provide positive reinforcement for caregivers’ BF (e.g., psychological support, peer support, and helping them rebuild life goals), as this may help reduce depression in both caregivers and individuals with schizophrenia. Additionally, assessing suicide risk in both groups is essential for preventing suicides among individuals with schizophrenia and their caregivers alike.

## Figures and Tables

**Table 1:** Demographic and clinical characteristics in schizophrenia patients and caregivers at 6-month follow-up (N = 144).

	Patients N (%), N = 72	Caregivers N (%), N = 72	Total N (%), N = 144	Pair t/χ^2^	*p*
Gender				1.08	0.30
Male	23 (31.9)	29 (40.3)	52 (36.1)		
Female	49 (68.1)	43 (59.7)	92 (63.9)		
Age, years mean	48.1 ± 11.1	60.4 ± 12.8	54.2 ± 13.5	−6.16	<0.001
Age of onset	29.0 ± 12.2				
Duration of schizophrenia	19.1 ± 10.4				
Duration of caring		16.6 ± 9.5			
Years of education	12.5 ± 3.5	12.4 ± 3.3	12.4 ± 3.4	0.20	0.85
Education				0.67	0.41
Less than high school (<12)	13 (18.1)	17 (23.6)	30 (20.8)		
More than college (≥12)	59 (81.9)	55 (76.4)	114 (79.2)		
Marital Status				21.78	<0.001
Unmarried	50 (69.4)	22(30.6)	72 (50.0)		
Married	22 (30.6)	50(69.4)	72 (50.0)		
Unemployment	58 (80.6)	43(59.7)	101 (70.1)	7.46	0.006
Comorbid with other diseases	44 (61.1)	39 (54.2)	83 (57.6)	0.71	0.40
Self-labeling psychiatric history	70 (98.6)	12 (16.9)	82 (57.7)	99.01	<0.001
No psychiatric history	1 (1.4)	60 (83.3)	61 (42.4)		
Schizophrenia	45 (62.5)	1 (1.4)	46 (31.9)		
Depressive disorder	6 (8.3)	8 (11.1)	14 (9.7)		
Insomnia	8 (11.1)	3 (4.2)	11 (7.6)		
Anxiety disorder	3 (4.2)	3 (4.2)	6 (4.2)		
Panic disorder	4 (5.6)	1 (1.4)	5 (3.5)		
Bipolar disorder	3 (4.2)	0	3 (2.1)		
Obsessive–compulsive disorder	2 (2.8)	0	2 (1.4)		
Suicide history	20 (27.8)	2 (2.8)	22 (15.3)	17.38	<0.001
Anxiolytics/Hypnotics use	50 (69.4)	12 (16.7)	62 (43.1)	40.90	<0.001
Family psychiatric history				10.12	0.001
No psychiatric history	49 (68.1)	30 (41.7)	79 (54.9)		
Schizophrenia	6 (8.3)	36 (50.0)	42 (29.2)		
Depressive disorder	8 (11.1)	4 (5.6)	12 (8.3)		
Anxiety disorder	0	2 (2.8)	2 (1.4)		
Bipolar disorder	2 (2.8)	0	2 (1.4)		
Insomnia	3 (4.2)	0	3 (2.1)		
Dementia	2 (2.8)	0	2 (1.4)		
Family suicide history	3 (4.2)	6 (8.3)	9 (6.3)	1.07	0.30
NPRS	2.3 (0–9)	2.7 (0–10)	2.5 (0–10)	−0.98	0.33
TDQ	9.8 (0–29)	8.9 (0–38)	9.3 (0–38)	0.67	0.50
APGAR	8.3 ± 3.0	7.1 ± 3.2	7.7 ± 3.1	2.34	0.021
BFI-10					
Extraversion	4.5 ± 2.0	5.3 ± 1.9	4.9 ± 2.0	−2.37	0.019
Agreeableness	6.0 ± 1.5	6.4 ± 1.6	6.2 ± 1.5	−1.58	0.12
Conscientiousness	6.6 ± 1.8	7.6 ± 1.6	7.1 ± 1.8	−3.58	<0.001
Neuroticism	6.6 ± 1.9	5.2 ± 1.7	5.9 ± 2.0	4.76	<0.001
Openness	5.6 ± 2.1	6.3 ± 2.1	5.9 ± 2.1	−1.86	0.06
LTEQ	0.8 (0–9)	0.7 (0–12)	0.7 (0–12)	0.25	0.80
BFS total scores	53.2 ± 20.2	64.9 ± 21.7	58.0 ± 21.7	−3.36	0.001
Acceptance	8.1 ± 3.7	9.4 ± 3.5	8.7 ± 3.6	−2.24	0.027
Family Relations	5.1 ± 2.3	6.2 ± 2.2	5.7 ± 2.3	−2.79	0.006
World View	8.3 ± 4.1	11.7 ± 4.3	10.0 ± 4.5	−4.80	<0.001
Personal Growth	15.1 ± 7.4	20.3 ± 8.1	17.7 ± 8.2	−3.98	<0.001
Social Relations	7.9 ± 3.8	8.2 ± 3.6	8.0 ± 3.7	−0.59	0.56
Health Behavior	8.7 ± 3.9	9.2 ± 3.6	8.9 ± 3.7	−0.80	0.42
EMIC Stigma Scale	14.0 ± 11.3	9.1 ± 7.9	11.5 ± 10.0	3.03	0.003
MINI suicidality	1.4 (0–17)	0.6 (0–23)	1.0 (0–23)	1.55	0.12
MINI suicidality level				24.27	<0.001
High risk	1 (1.4)	2 (2.8)	3 (2.1)		
Low risk	23 (31.9)	1 (1.4)	24 (16.7)		
No risk	48 (66.7)	69 (95.8)	117 (81.3)		
SAS total scores	12.4 (0–52)	13.4 (0–52)	12.9 (0–52)	−0.49	0.63
Negative ideation	1.8 (0–15)	1.9 (0–20)	1.9 (0–20)	−0.21	0.84
Positive ideation	6.4 (0–20)	7.0 (0–20)	6.7 (0–20)	−0.58	0.56
Impulsivity	1.1 (0–11)	2.0 (0–15)	1.6 (0–15)	−2.14	0.034
Aggression	3.1 (0–18)	2.5 (0–16)	2.8 (0–18)	0.98	0.33

Values are presented as number (%) or mean ± standard deviation (range). NPRS = Numeric Pain Rating Scale; APGAR = Family APGAR Index; TDQ = Taiwanese Depression Questionnaire; BFI-10 = Big Five Inventory-10; LTEQ = Brief Life Event Questionnaire; BFS = Benefit Finding Scale; EMIC = Explanatory Model Interview Catalogue; MINI = Mini International Neuropsychiatric Interview; SAS = Suicide Assessment Scale.

**Table 2:** Psychiatric diagnoses in schizophrenia patients and caregivers at 6-month follow-up (N = 142).

MINI Diagnosis	Patients, N = 72	Caregivers, N = 72
Depressive disorders	2 (2.8)	21 (29.2)
Major depressive disorder	0	6 (8.3)
Other specified depressive disorder	2 (2.8)	13 (18.1)
Persistent depressive disorder	0	2 (2.8)
Anxiety disorders	2 (2.8)	8 (11.1)
Generalized anxiety disorder	0	6 (8.3)
Panic disorder	0	2 (2.8)
Obsessive–compulsive disorder	2 (2.8)	0
Insomnia disorder	0	6 (8.3)
Schizophrenia	72 (100.0)	2 (2.8)
Alcohol use disorder	0	1 (1.4)
No diagnosis	0	41 (56.9)

MINI = Mini International Neuropsychiatric Interview. Values are presented as number (%).

**Table 3:** Pearson correlation analysis for patients’ BF, stigma, suicide risk, and depression with caregivers’ BF, stigma, suicide risk, and depression.

		Patient				Caregiver		
Variable	TDQ	BFS	SAS	EMIC	TDQ	BFS	SAS	EMIC
Patient-TDQ	1							
Patient-BFS	−0.403 ***	1						
Patient-SAS	0.633 ***	−0.470 ***	1					
Patient-EMIC	0.299 *	−0.272 *	0.392 **	1				
Caregiver-TDQ	0.356 **	−0.342 **	0.304 **	0.152	1			
Caregiver-BFS	−0.276 *	0.465 ***	−0.193	−0.195	−0.486 ***	1		
Caregiver-SAS	0.377 **	−0.308 **	0.388 **	0.252 *	0.762 ***	−0.330 **	1	
Caregiver-EMIC	−0.134	0.042	−0.034	0.270 *	0.405 ***	−0.201	0.474 ***	1

Note: TDQ = Taiwanese Depression Questionnaire; BFS = Benefit Finding Scale; SAS = Suicide Assessment Scale; EMIC = Explanatory Model Interview Catalogue; * Correlation is significant at 0.05 level; ** Correlation is significant at 0.01 level; *** Correlation is significant at 0.001 level.

## Data Availability

The data of the current study are available from the corresponding author on reasonable request.
